# Dual-Valence Copper Nanostructures with Cu^+^/Cu^2+^ Interfaces for High-Sensitivity Glucose Electrochemical Sensing

**DOI:** 10.3390/nano14242000

**Published:** 2024-12-13

**Authors:** Zhipeng Yu, Pengxu Yan, Yilei Sheng, Chengwei Zhang, Zhun Qiao, Qikui Fan, Chuncai Kong, Zhimao Yang

**Affiliations:** 1 Ministry of Education Key Laboratory for Non-Equilibrium Synthesis and Modulation of Condensed Matter, Shaanxi Province Key Laboratory of Advanced Functional Materials and Mesoscopic Physics, School of Physics, Xi’an Jiaotong University, Xi’an 710049, China; 2 Xi’an Rare Metal Materials Institute Co., Ltd., Xi’an 710016, China

**Keywords:** dual-valence copper composite, interfaces, metal oxide, glucose, detection

## Abstract

Copper-based materials, renowned for their redox versatility and conductivity, have extensive applications in electrochemical sensing. Herein, we construct stable Cu^+^/Cu^2+^ interfaces within dual-valence copper nanostructures to achieve enhanced sensitivity in glucose sensing. By employing a hydrolysis method to tune Cu^2+^/Cu^+^ ratios precisely, we achieved an optimal electrochemical interface with heightened stability and reactivity. The Cu^+^/Cu^2+^ interface-based flexible electrode demonstrated excellent glucose sensitivity (332.4 µA mmol/L^−1^ cm^−2^ at +0.65 V), wide linear range (up to 10 mmol), a low detection limit of 1.02 nmol/L, and strong selectivity, including detection in human sweat, making this study significant for advanced electrochemical sensors.

## 1. Introduction

Copper-based materials, characterized by versatile redox states [1,2,3] and excellent conductivity [4,5,6], have gained prominence in electrochemical sensing [7,8]. The synergistic effects between Cu^+^/Cu^2+^ ions at the Cu^+^/Cu^2+^ interface enhance adsorption [9], activation [10], and electron transfer [11], providing high sensitivity [12] and selectivity [13] in sensors, notably for glucose [7,14,15]. The Cu^+^/Cu^2+^ interface enables effective electron transport, where glucose molecules reduce Cu^2+^ to Cu^+^ upon interaction, generating a measurable current signal. Traditional enzyme-based glucose sensors face limitations in stability; thus, our non-enzymatic Cu^+^/Cu^2+^ interface offers a promising alternative for glucose detection [14,16,17]. This work employs cuprous chloride (CuCl) triangular prisms as templates and a controlled hydrolysis approach to achieve stable, high-activity Cu^+^/Cu^2+^ interfaces with optimized electrochemical properties for glucose sensing.

In electrochemical sensors, the redox cycling of the Cu^+^/Cu^2+^ interface underpins selective recognition of target molecules [7,15,18]. Constructing a stable Cu^+^/Cu^2+^ interface can significantly improve both sensor sensitivity and response speed [19,20]. For instance, in non-enzymatic glucose detection, the Cu^+^/Cu^2+^ interface can function as an electron-transport channel, whereby the interaction of glucose molecules with Cu^2+^ sites on the electrode surface triggers electron transfer, reducing Cu^2+^ to Cu^+^ [21,22,23]. This electron transfer can be directly measured as a current signal to detect glucose concentration, offering a stable alternative to traditional enzyme-based sensors [14,24]. This mechanism has been applied to materials such as Ni-based [7], ZnO-Cu*_x_*O nanostructures [25], and TiO_2_ nanotube arrays [26].

However, challenges remain in the controlled preparation of Cu^+^/Cu^2+^ interfaces [15,27]. In this work, we employ cuprous chloride triangular prism particles as a template, using the hydrolysis properties of cuprous chloride and ascorbic acid reduction properties to construct a nanostructure with a well-defined Cu^+^/Cu^2+^ interface. This method allows precise tuning of the Cu^+^/Cu^2+^ ratio, resulting in an interface with enhanced electrochemical stability and reactivity. Additionally, by adjusting reaction conditions, the distribution and structure of the Cu^+^/Cu^2+^ interface can be optimized, significantly improving its performance in sensing applications.

This study aims to finely control the formation and structural features of the dual-valence copper composite (DVC-Cu) interface, specifically the Cu^+^/Cu^2+^ interface, to achieve an enhanced electrochemical response. Our results indicate that constructing a DVC-Cu interface not only improves selectivity in electrochemical reactions but also significantly increases the sensitivity of flexible electrochemical sensors. Under a steady-state test with a small volume of electrolyte (30 µL, 332.4 µA mmol/L^−1^ cm^−2^), flexible electrodes utilizing the Cu^+^/Cu^2+^ interface as the active material demonstrated excellent sensitivity for glucose detection at an optimal voltage of +0.65 V achieving a low detection limit of 1.02 nmol/L, with notable selectivity and stability. Additionally, the sensor displayed promising performance in glucose detection from human sweat. Through optimized control of interface morphology and electronic structure, this study reveals the underlying mechanisms of the Cu^+^/Cu^2+^ interface, providing a theoretical foundation for the development of efficient electrochemical materials and sensors.

## 2. Materials and Methods

### 2.1. Materials

CuCl_2_·2H_2_O, anhydrous ethanol, D-glucose, ascorbic acid (AA), uric acid, urea, NaCl, lactic acid, Nafion, K_4_Fe(CN)_6_, K_3_[Fe(CN)_6_], and isopropanol were all analytical reagent (AR) grade and were purchased from Aladdin Reagent Co., Ltd. (Shanghai, China) without further purification. Synthetic sweat and saliva were purchased from Tianjin Huasheng Chemical Reagent Co., Ltd. (Tianjin, China) without further purification. The preparation method and a schematic of the test electrode are illustrated in Figure S1.

### 2.2. Synthesis of CuCl NPs

To prepare CuCl nanoparticles, a 100 mL 0.2 mol/L aqueous solution of CuCl_2_·2H_2_O and a 100 mL 0.4 mol/L aqueous solution of AA were prepared separately. The ascorbic acid solution was placed on a magnetic stirrer and stirred at 500 rpm for 2 min. The CuCl_2_·2H_2_O solution was then slowly added to the 100 mL ascorbic acid solution under continuous stirring. The reaction was allowed to proceed for 30 min, after which the mixture was left to stand for 30 min to settle. The supernatant was then removed, and the remaining solution was centrifuged at 8000 rpm for 15 min. The precipitate was washed with ethanol and centrifuged again at 8000 rpm for 15 min, repeating this washing and centrifugation process two additional times. After washing, the final sample was freeze-dried in a vacuum freeze dryer for 12 h, then sealed and labeled for further use.

### 2.3. Synthesis of Cu_2_O NPs

Aqueous solutions of 0.1 mol/L CuCl_2_·5H_2_O, 0.1 mol/L NaOH, and 0.1 mol/L AA were prepared in separate beakers. The CuCl_2_·5H_2_O solution was placed on a magnetic stirrer and dispersed at 500 rpm for 10 min. The NaOH solution was then slowly added to the stirring CuCl_2_·5H_2_O solution, followed by the slow addition of the AA solution. The mixture was stirred continuously for an additional 30 min. After the reaction was complete, the product was allowed to precipitate naturally at room temperature. The supernatant was carefully removed, and the remaining suspension was centrifuged at 8000 rpm for 15 min, with the supernatant discarded afterward. The precipitate was washed by centrifuging with deionized water three times. The cleaned sample was then freeze-dried for 12 h. Finally, the sample was collected, sealed, labeled, and stored for subsequent use.

### 2.4. Synthesis of DVC-Cu

As illustrated in Figure S1, 0.1 g of CuCl particles was weighed and added to 200 mL of deionized water. The mixture was stirred at 25 °C, 500 rpm for 5 min. The CuCl suspension was then transferred to a stable water bath maintained at 50 °C, where it was stirred at 500 rpm for an additional 30 min. After completion of the reaction, the suspension was allowed to naturally precipitate at 25 °C for 1 h. The reaction mixture was then collected, and the supernatant was isolated by centrifugation at 8000 rpm for 15 min. The collected material was washed by repeating centrifugation with deionized water (18.2 MΩ·cm, 23 °C) at 8000 rpm two more times. Finally, the prepared sample was freeze-dried in a freeze dryer for 12 h, after which it was sealed (atmosphere glove box, Ar), labeled, and stored (atmosphere glove box, Ar) for further use.

### 2.5. Testing Methodology

All electrochemical measurements were conducted using a three-electrode electrochemical system, which consisted of a carbon-coated working electrode, an Ag/AgCl-coated reference electrode, and a carbon-coated substrate electrode, with the prepared sample serving as the active material. It is worth noting that the working electrode diameter was 0.3 cm. The electrolyte solution was a 30 µL mixture of 0.1 mol/L NaOH and 0.1 mol/L NaCl. Cyclic voltammetry (CV) measurements were performed within a potential range from 0 V to +0.7 V. For electrochemical characterization, an electrolyte solution containing 0.1 mol/L KCl, 0.1 mol/L K_3_[Fe(CN)_6_], and 0.1 mol/L K_4_[Fe(CN)_6_] was used. In order to eliminate the effect of chloride ion concentration on sweat measurement concentration, synthetic sweat was configured as a mixed solution containing sodium chloride-saturated solution. During anthropometry, 1 g sodium chloride powder was applied to the subject’s epidermis during in situ measurement of human skin to ensure that the measured sweat was sodium chloride-saturated solution. A precision test paper is used to determine the pH value of the sweat secreted by the subject’s epidermis. Each error bar was obtained by statistical analysis of three sets of measurement data.

In skin testing, a standard three-electrode system was employed. The subject agrees to donate the test sample, the subject is 1 person, and the donor consent form is signed by himself according to the requirements of the clinical and ethics committee. A flexible film was inserted into an electrochemical workstation adapter (CHI-660e electrochemical workstation, Shanghai Chenhua, Shanghai, China), with the flexible device affixed to the surface of a conformal dressing. The skin-adherent dressing was applied to the outer upper arm of the subject. Prior to sensor data collection, the subject engaged in 15 min of physical activity to produce sweat samples. In order to avoid the interference of temperature on the measurement results, the measurement of synthetic sweat was set at 37 °C, and the skin temperature of the tested sample was also maintained at 37 °C by controlling the temperature of the air conditioner. The finger blood’s glucose concentration was measured by a commercial blood glucose meter (glucose meter 590, Jiangsu Yuyue Medical Equipment & Supply Co., Ltd., Danyang, China). The phase composition of the sample was characterized using X-ray powder diffraction (XRD, Bruker-AXS D8 ADVANCE diffractometer, Billerica, MA, USA, Cu kα = 0.1506 nm), while the microstructure and morphology were analyzed with scanning electron microscopy (SEM, JEOL, Tokyo, Japan, JSM-7000F field-emission scanning electron microscope, 15 kV), transmission electron microscopy (TEM, JEOL, JEM-2100 transmission electron microscope, AC 120 kV), and X-ray photoelectron spectroscopy (XPS, Thermo Fisher ESCALAB Xi+, Al Kα, Waltham, MA, USA).

## 3. Results and Discussion

### 3.1. Structural and Morphological Analysis

**Figure 1:**
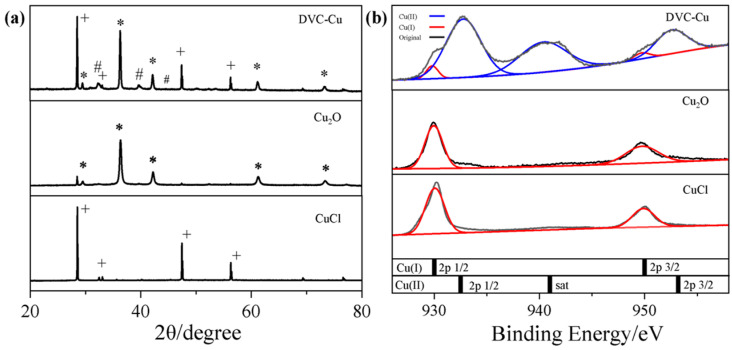
(**a**) XRD patterns and (**b**) XPS Cu 2p spectra of CuCl, Cu_2_O, and DVC-Cu.

Figure 1a presents the XRD pattern of the sample. As shown in Figure 1a, the characteristic peaks of the CuCl precursor align with the standard PDF card ICDD PDF No. 00-006-0344 (marked as +). In comparison, the XRD spectrum of the reference sample, Cu_2_O, matches the PDF standard card ICDD PDF No. 01-077-0199 (marked as *). The hydrolytic reduction of CuCl plays a key role in the synthesis of DVC-Cu. During this process, water molecules interact with the CuCl precursor, gradually converting the compound into intermediate products and Cu_2_O. The intermediate CuO·3H_2_O formed through hydrolytic reduction shows characteristic peaks that correspond to the PDF standard card ICDD PDF No. 00-036-0545 (marked as #). Additional characteristic peaks appear at 29.6°, 36.5°, 42.4°, and 73.7°, corresponding to the (110), (111), (200), and (311) crystal planes of Cu_2_O, respectively. Thus, after hydrolytic reduction, the CuCl sample transforms into DVC-Cu, exhibiting a structure with a CuCl trigonal core and an external layer comprising CuO·3H_2_O and Cu_2_O.

The mechanism of hydrolytic reduction can be explained through the stepwise reaction process of CuCl. With a sufficient concentration of ascorbic acid, the strong reducing nature of ascorbic acid stabilizes the CuCl precursor, preventing hydrolytic reduction. However, after repeated centrifugation and washing with deionized water, the ascorbic acid concentration and its protective reducing effect decreased significantly, initiating the hydrolytic reduction of CuCl. Water acts as a reactant, breaking the Cu–Cl bond (r_Cu-Cl_ ≈ 2.2 Å) [28,29,30] and forming the Cu–O bond (r_Cu-O_ ≈ 1.92 Å) [31,32,33], resulting in the formation of copper oxides. Considering that CuO·3H_2_O, an intermediate containing Cu^2+^, forms during the hydrolytic reduction, the acidic byproduct HCl creates a low-pH environment that accelerates the conversion, further promoting the formation of copper chloride species. While centrifugation removes residual ascorbic acid from the CuCl precursor, some ascorbic acid molecules remain adsorbed on the CuCl particle surface, reducing free Cu^2+^ to Cu_2_O. When the outer Cu_2_O layer reaches sufficient thickness, it hinders further conversion of the CuO·3H_2_O intermediate, ultimately forming a CuCl core with an outer layer of Cu_2_O and minor CuO·3H_2_O, resulting in a structure characterized by a Cu⁺/Cu^2^⁺ interface.

To confirm the elemental valence states, XPS measurements were conducted. Figure 1b shows the XPS spectra of CuCl, Cu_2_O, and DVC-Cu. The Cu 2p spectra indicate characteristic binding energies of 932.1 eV and 952.4 eV for CuCl, corresponding to the Cu^+^ state in the Cu 2p_1/2_ and Cu 2p_3/2_ orbital [34], suggesting that Cu in the CuCl precursor is in the Cu⁺ state with no presence of Cu^2^⁺. For the Cu_2_O reference sample, Cu 2p peaks at 932.6 eV and 952.4 eV indicate the presence of both Cu^+^ states. In the hydrolytically reduced DVC-Cu, peak deconvolution of the XPS spectrum reveals two sets of characteristic peaks at 932.0 eV and 952.0 eV, as well as peaks at 932.4 eV, 939.7 eV, and 952.3 eV, indicating the coexistence of Cu^+^ and Cu^2+^ states, further confirming the presence of a Cu^2+^/Cu^+^ interface. Combined XPS and XRD analyses demonstrate that CuCl predominantly exists in the Cu(I) state, while DVC-Cu contains both Cu^+^ and Cu^2+^ states. The presence of Cu^2+^ in the surface structure of DVC-Cu is particularly important, suggesting that the hydrolytic reduction process led to the formation of a Cu^+^/Cu^2+^ interfacial structure.

**Figure 2:**
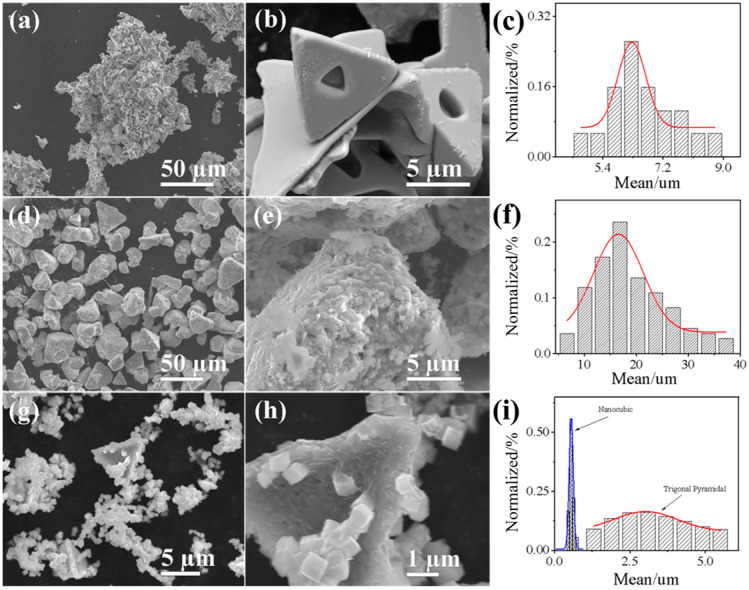
FE-SEM images and particle size distribution of (**a**–**c**) CuCl, (**d**–**f**) Cu_2_O, and (**g**–**i**) DVC-Cu.

Figure 2 shows the morphological characteristics of the samples as observed via field-emission scanning electron microscopy (FE-SEM). The FE-SEM images reveal that CuCl exhibits a smooth trigonal pyramidal structure with an average particle size of approximately 6.3 µm (Figure 2a–c). As shown in Figure 2d–f, the Cu_2_O sample has an irregular polyhedral shape with a slightly larger particle size of about 16.5 µm. For the DVC-Cu sample (Figure 2g–i), the overall particle size is significantly smaller, with an average size of 3.1 µm, and the outer nanocubic structures have dimensions around 570 nm. This reduction in size results from the hydrolytic reduction process, which involves the hydrolytic reaction between CuCl and water, where the outer layer of CuCl particles undergoes hydrolytic reduction, forming smaller Cu_2_O nanostructures. The uniformity of the nanocubic structures and the noticeable decrease in particle size indicate that hydrolytic reduction effectively reconfigures CuCl into more homogeneous DVC-Cu.

The elemental composition of these samples was further analyzed using energy-dispersive spectroscopy (EDS) to investigate how the hydrolytic reduction process affects the distribution of elements such as Cu, Cl, and O. As shown in Figure S2, EDS analysis of the CuCl sample reveals that the distribution patterns of Cu and Cl are consistent with the trigonal pyramidal shape observed in the FE-SEM images. For DVC-Cu, EDS analysis shows a marked decrease in Cl distribution, indicating a significant change in elemental distribution within the EDS measurement range. The Cl concentration is notably reduced in the outer nanocubic particles, appearing as distinct squares in the EDS-Cl map, while the trigonal core retains a higher Cl concentration. This retention is attributed to the limited depth of hydrolytic reduction during DVC-Cu formation, resulting in residual Cl ions within the trigonal core that are detectable. Despite these depth limitations, the hydrolytic reduction process effectively converts a significant amount of Cu^2+^ to Cu_2_O. Compared to the pure CuCl sample, the diminished Cl signal in the hydrolytically reduced sample aligns with the expected outcomes of the hydrolytic reduction mechanism. This further supports the presence of a Cu^+^/Cu^2+^ interface within DVC-Cu.

Combined analysis of XRD, FE-SEM, and FESEM-EDS results suggests that significant structural changes occur in CuCl during the hydrolytic reduction process. These transformations lead to the formation of DVC-Cu with interfacial structures, transitioning from bulk particles to an interface-rich configuration, which enhances its structural properties and potential for various applications.

**Figure 3:**
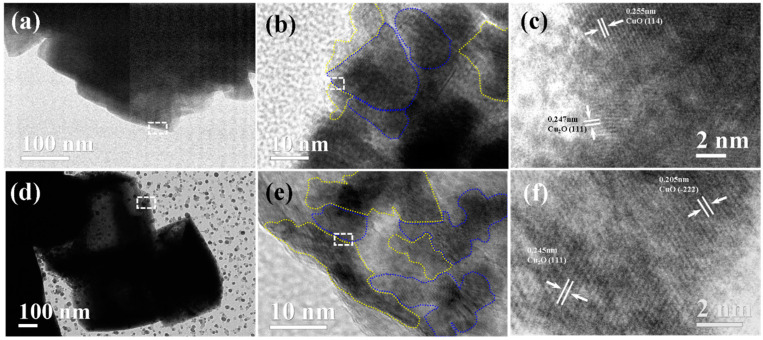
(**a**) TEM, (**b**) HRTEM, and (**c**) detailed lattice structures images of the DVC-Cu boundary, (**d**) TEM, (**e**) HRTEM, and (**f**) detailed lattice structures images of the hollow cubic structures in the outer layer. White dashed boxes in the figure were the observed position of next serial number image.

Figure S3 illustrates the boundary features of DVC-Cu via TEM, along with the boundary of CuCl particles. The boundary angle of the CuCl nanopyramids is approximately 120° with a smooth surface, consistent with the trigonal pyramidal morphology observed in FESEM images. The edge tangents of the DVC-Cu core closely resemble those of the original CuCl, while the outer layer exhibits a distinct multi-cubic, angular shape, indicating that the boundary interface of CuCl has changed (Figure 3a). This geometric transformation is a direct result of the hydrolytic reduction of CuCl, forming DVC-Cu and significantly increasing the surface area.

Further examination of the DVC-Cu boundary reveals that the particles predominantly exhibit polyhedral morphology with larger particle sizes and polyhedral boundaries. High-resolution transmission electron microscopy (HRTEM) analysis was conducted to investigate the external structure of DVC-Cu in more detail. Figure 3b,c show high-resolution boundary images of DVC-Cu. The lattice-measured regions for Cu^2+^ and Cu^+^ are marked with yellow and blue dashed lines in Figure 3b. Cu^+^ is present in both the outer and inner layers, attributed to the hydrolytic reduction process of CuCl. The HRTEM image in Figure 3c displays lattice spacings of 0.255 nm and 0.247 nm, corresponding to the (114) plane of CuO·3H_2_O and the (111) plane of Cu_2_O, respectively. Figure 3d and Figure S4a–c present TEM images of hollow cubic particles in the outer layer of DVC-Cu. These hollow cubes, smaller and more uniform than the CuCl sample, are further loaded with small particles on their surface. The hollow Cu_2_O cubes surrounding DVC-Cu measure approximately 570 nm with a shell thickness of about 54 nm (Figure S4b), while the small particles have an average size of 6 nm (Figure S4c,d). The hydrolytic reduction process effectively converts CuCl into smaller, more uniform particles. The HRTEM image of the hollow cube is shown in Figure 3e. The overlapping distribution of Cu^2+^ and Cu^+^ near the outer regions is more prominent than in the core, as the free Cu^2+^ in the solution grows more rapidly than the Cu^2+^ adsorbed on the surface, indicating abundant Cu^2+^/Cu^+^ interfacial structures in DVC-Cu. Figure 3f presents a magnified HRTEM image of the boundary of the outer-layer hollow cube in DVC-Cu. Lattice spacings of 0.205 nm and 0.245 nm are observed, corresponding to the (-222) plane of CuO·3H_2_O and the (111) plane of Cu_2_O, respectively. Both the core boundary and the boundary of the hollow nanocubic particles exhibit lattice spacings corresponding to CuO·3H_2_O and Cu_2_O, demonstrating that Cu^2+^/Cu^+^ generated in the hydrolytic reduction process are structurally consistent across phases. The HRTEM image of the small particles on the surface of the hollow cube (Figure S4e) shows a lattice spacing of 0.245 nm, corresponding to the (111) plane of Cu_2_O, indicating that free ascorbic acid effectively reduces free Cu^2+^. The gradient distribution of Cu^2+^ on the DVC-Cu boundary and hollow cube surface further supports the hydrolytic reduction mechanism proposed for DVC-Cu formation, where Cu^2+^ concentration decreases progressively, and the boundaries of DVC-Cu and hollow nano-cubes remain exposed. To further verify the crystal structure, electron diffraction was performed using TEM, as shown in Figure S4f. The diffraction pattern displays a polycrystalline structure characterized by multiple diffraction points, with polycrystalline rings dominating the pattern, indicating a range of orientations within the crystal structure. This multi-crystallinity is a hallmark of DVC-Cu, suggesting that the hydrolytic reduction process facilitates the formation of crystals with various orientations.

In conclusion, the HRTEM and TEM results indicate structural changes at the interface during the hydrolytic reduction of CuCl, leading to the formation of DVC-Cu. The creation of a Cu⁺/Cu^2^⁺ interfacial structure is a direct consequence of controlling DVC-Cu formation through hydrolytic reduction.

### 3.2. Electrochemical Analysis

**Figure 4:**
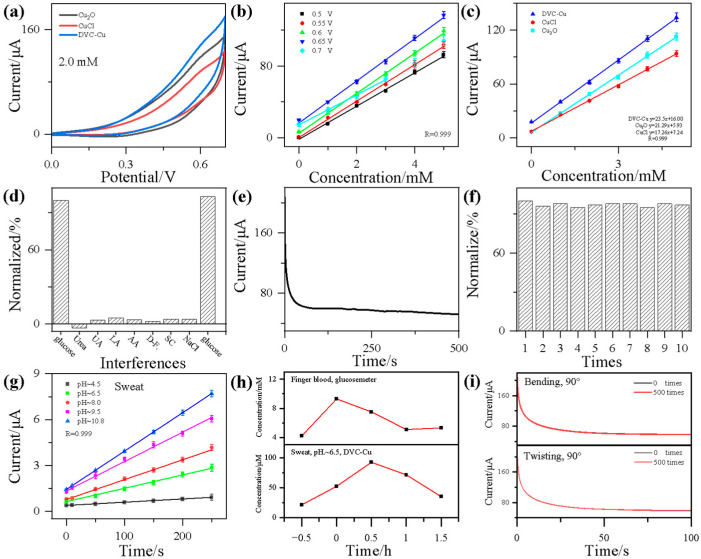
(**a**) CV curves of CuCl, Cu_2_O, and DVC-Cu materials at a glucose concentration of 2.0 mmol/L. (**b**) Relationship between glucose concentration and response current for DVC-Cu material under different measurement voltages. (**c**) Glucose concentration vs. response current for various samples at a measurement potential of +0.65 V. (**d**) Normalized interference effects of interferents. (**e**) I-T response curve for continuous stability measurement over 500 s at a glucose concentration of 2.0 mmol/L and (**f**) normalized curve for 10 repeated measurements. (**g**) Glucose concentration vs. response current for various pH of synthetic human sweat at a measurement potential of +0.65 V. (**h**) Real sweat and finger blood’s glucose response current before and after meals by commercial blood glycosometer and DVC-Cu sensor. (**i**) I-T response curve before and after 500 cycles of bending and twisting.

DVC-Cu was composed of Cu(II) oxide (CuO) and Cu(I) oxide (Cu_2_O). The glucose sensing mechanism involves a mono-electron reaction based on the Cu^+^/Cu^2+^ redox couple, where these ions act as intermediates, facilitating the transformation of copper ions during the sensing process. Electrons were released from glucose and transferred through the copper ion to the current collector. The active sites and electrochemical surface area (ECSA) of Cu_2_O and CuCl were insufficient for effective glucose sensing. The incorporation of Cu^2+^ in DVC-Cu enhances the sensing process. DVC-Cu exhibits superior non-enzymatic glucose sensing performance compared to Cu_2_O and CuCl. To investigate the glucose electrochemical sensing performance of the synthesized DVC-Cu, electrodes integrated with CuCl, Cu_2_O, and DVC-Cu as active materials were tested and compared. Figure S5 and Figure 4a display the cyclic voltammetry (CV) curves at a scan rate of 50 mV s^−1^ in a mixed electrolyte of 0.1 mol/L NaOH and 0.1 mol/L KCl with glucose concentrations of 0 mmol/L and 2.0 mmol/L. The CV curves at different glucose concentrations reveal an increase in current response with rising glucose concentration (Figure S6a–c). Among the electrodes, the DVC-Cu electrode exhibited the highest CV response current, while the CuCl electrode showed the lowest, with oxidation peaks in the range of +0.5 V to +0.7 V.

Furthermore, to compare glucose sensing performance at different potentials, current-time (i-t) curves were recorded for various glucose concentrations at potentials of +0.5 V, +0.55 V, +0.6 V, +0.65 V, and +0.7 V, as shown in Figure S7, with fitted i-t response curves illustrated in Figure 4b. In Figure S8, the linear range of the sensor is measured (up to 10 mmol/L). The relationship between the response current intensity j and the substrate concentration [S] was described by the Michaelis–Menten equation, j = K_cat_ × [S]/(K_m_ + [S]), where K_m_ and K_cat_ are the Michaelis constant and the apparent catalytic turnover rate, respectively. These parameters were determined experimentally. If the glucose concentration is sufficiently low and K_m_ is much larger than [S], the [S] term becomes negligible, leading to a linear relationship between the response current intensity j and glucose concentration in the electrolyte. As the glucose concentration increases and [S] becomes large enough, the response current intensity j follows a nonlinear relationship with glucose concentration. For the DVC-Cu-based sensor, the linear fitting equation is I = 16.0 + 23.5 × C (mmol/L), where C is the glucose concentration. The sensitivity of the sensor was calculated as k/s, where k is the slope of the linear fitting equation, and s is the electrode area. Based on the equation and experimental data, we determined that k = 23.5 µA mmol/L^−1^ and s = 0.0707 cm^−2^, resulting in a sensitivity of k/s = 332.4 µA mmol/L^−1^ cm^−2^. The detection limit of the sensor was found to be 1.02 nmol/L, calculated using the formula 3σ/s, where σ/s was the standard deviation of the background current and s was the slope of the calibration curve. The response current intensity increased with glucose concentration (Table S1). Additionally, amperometric (i-t) tests were conducted to evaluate the glucose sensing performance of the three electrodes at +0.65 V in mixed electrolytes with varying glucose concentrations, as shown in Figure S6d–f and Table S2. The DVC-Cu electrode displayed the highest response current, while the pure CuCl electrode showed poor performance (Figure 4c). Due to the Cu^+^/Cu^2+^ interface and special morphological features, DVC-Cu shows lower LOD and higher sensing ability than that of CuCl and Cu_2_O.

Interference resistance is a critical parameter in assessing glucose sensing selectivity. To evaluate this, 1.0 mmol/L of potential interferents such as urea, uric acid (UA), lactic acid (LA), ascorbic acid (AA), D-fructose (D-F.), and sucrose (SC) were added to the system. After first introducing 2.0 mmol/L glucose electrolyte, 30 µmol/L of 2.0 mmol/L glucose and 5.0 mmol/L interferent electrolytes were measured, followed by a repeat glucose measurement for comparison. As shown in Figure S9a, the response current from interferents was significantly weaker compared to glucose. Figure 4d provides a clearer visualization of the impact of interferents on the electrode. With the glucose current response set to 100%, the highest current response from any interferent was only 5.3%, confirming the strong selectivity of DVC-Cu.

Stability is another key parameter for evaluating electrochemical performance. Figure 4e shows the current response over 500 s after adding 2.0 mmol/L glucose. The signal remained stable without significant decline after 500 s. Figure 4f presents the electrode’s long-term stability, with continuous testing of the same electrode under identical conditions over 10 trials. While there was a slight decrease in response current in routine tests, the maximum current decay was only 5.2%, and after 10 cycles, the current retained 95.6% of its initial value.

To validate the practical performance of the prepared electrode, glucose concentration in various pH values of synthetic human sweat was measured, as shown in Figure 4g and Figure S10. As we know, the pH value of human sweat was not a constant value, depending on the body state of the body. Therefore, we selected synthetic human sweat with pH values of 4.5, 6.5, 8.0, 9.5, and 10.8 as the object of discussion, which can include acidic, neutral, and alkaline human sweat states. As Figure 4g shows, the pH value of human sweat was a key factor in glucose concentration vs. response current. First, the pH value of the subject’s sweat was determined according to the precision pH test paper, and the pH value of the secreted sweat was about 6.5 by comparison with the colorimetric card. This is due to the daily eating habits, physical status and sweat gland characteristics of the sample. After conversion by the calibration curve, the glucose content in the sweat of the subject at −0.5 h, 0 h, +0.5 h, and +1.5 h of food intake were 21.3 µmol/L, 52.3 µmol/L, 92.6 µmol/L, 71.6, and 35.0 µmol/L, respectively. As indicated in Figure 4h and Figure S9b, before and after food intake, the glucose concentration in sweat showed a noticeable change, returning to baseline levels after approximately 1.5 h. To compare the reliability of the detection, blood glucose concentrations in fingertip blood were measured at the same time using a commercial glucose meter. The results showed that the blood glucose concentration in the finger blood also changed significantly, and the blood glucose concentration increased more rapidly than the glucose concentration in sweat.

In addition, in order to discuss whether the prepared glucose sensor also has the potential application of saliva glucose sensing function, the response current of synthetic saliva under different glucose concentration conditions was also measured (Figure S11). As shown in Figure S11, the sensor has sensing ability in saliva. Furthermore, the real human serums were performed by the prepared electrode, which calibrated by a biochemical analyzer were performed (Table S3). The sensor’s practicality for glucose detection was evaluated using human blood serum samples. As summarized in Table S3, glucose concentrations determined by an automatic biochemical analyzer were 4.75 mmol/L, 9.25 mmol/L, and 11.20 mmol/L, whereas the sensor yielded values of 4.98 mmol/L, 9.44 mmol/L, and 11.31 mmol/L, with relative standard deviations (RSD) of 5.25%, 3.13%, and 1.35%, respectively. The strong correlation between the results validates the sensor’s reliability for accurate glucose measurement in real serum samples. This demonstrates the efficacy of the fabricated glucose electrochemical sensor for glucose detection in sweat, saliva, and serum. Figure S12a,b shows physical images of the integrated electrode and sweat measurement setup.

To further assess the mechanical performance of the sensor, the sample was subjected to external twisting and bending (Figure S12c,d). Glucose electrochemical response current was measured before and after 500 cycles of twisting and bending (Figure 4i). The results indicate that DVC-Cu shows significant potential for application in the fabrication of electrochemical sensors.

**Figure 5:**
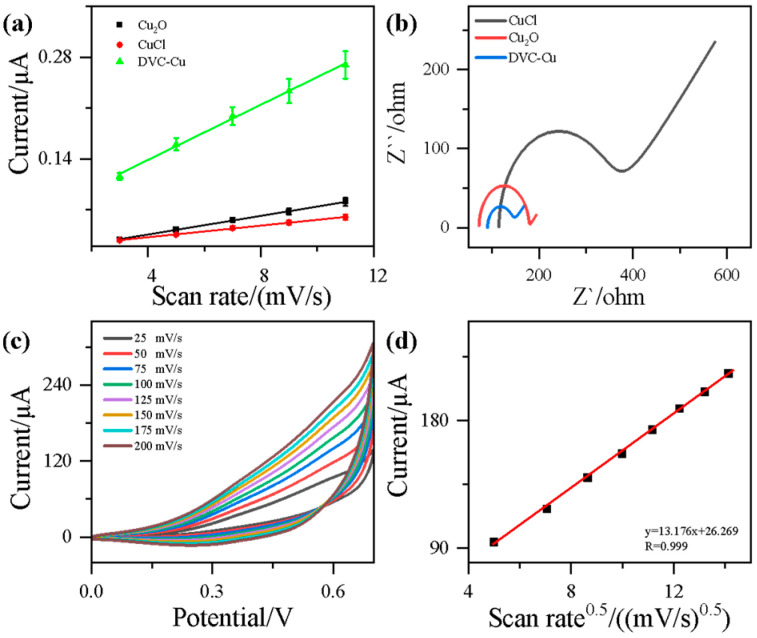
(**a**) ECSA-CV plot of DVC-Cu, (**b**) EIS spectrum, (**c**) CV curves at different scan rates under a 2.0 mmol/L glucose concentration, and (**d**) the relationship between the square root of the scan rate and response current at +0.65 V for DVC-Cu.

To analyze the electrochemical characteristics of DVC-Cu before and after its construction, cyclic voltammetry (CV) curves were recorded at different scan rates, as shown in Figure 5a and Figure S6g–i. The data in Figure 5a reveal that, in the absence of a redox process, the CV response current of the sample increases with higher scan rates, with DVC-Cu exhibiting the fastest growth rate. The electrochemical surface area (ECSA) of each sample was measured, showing a specific surface area of 0.091 µF·cm^2^ for CuCl, 0.057 µF·cm^2^ for Cu_2_O, and 0.269 µF·cm^2^ for DVC-Cu. This significant enhancement in electrochemical activity is primarily attributed to the Cu⁺/Cu^2^⁺ interface, which increases the surface area. Compared with CuCl and Cu_2_O, the Cu^+^/Cu^2+^ interface structure of DVC-Cu has an interface enhancement effect in more reaction sites at the interface of Cu^+^ and Cu^2+^ ion pairs because it has two valence structures of Cu^+^ and Cu^2+^. The hydrolytic reduction not only promotes the formation of an interface structure but also provides a larger electrochemically active area, which in turn facilitates electrochemical reactions. The increased surface area allows for more electron-transfer active sites, therefore improving overall electrochemical performance.

In comparative analyses, the DVC-Cu_x_O micro/nanostructure outperforms the CuCl and Cu_2_O control samples, exhibiting superior electrochemical properties. Figure 5b shows the electrochemical impedance spectroscopy (EIS) Nyquist plots obtained in a solution of 0.1 mol/L NaOH and 0.1 mol/L KCl. Comparing the impedance characteristics before and after DVC-Cu construction reveals a substantially lower electron-transfer resistance in DVC-Cu. This performance enhancement is likely due to the unique Cu⁺/Cu^2^⁺ interface structure, which facilitates efficient electron transfer. The Cu⁺/Cu^2^⁺ interface construction plays a crucial role here, as the increased surface area and porosity help reduce charge transfer resistance. Through fitting analysis, the resistance was determined to be 105.8 Ω for CuCl, 229.4 Ω for Cu_2_O, and significantly lower at 51.5 Ω for DVC-Cu.

Figure 5c,d illustrate the influence of different scan rates on the glucose oxidation behavior of DVC-Cu. When the scan rate increases from 25 mV/s to 200 mV/s, the oxidation peak current also increases, with a positive shift in potential. This behavior underscores the dynamic relationship between scan rate and electrochemical response, further validating the effectiveness of DVC-Cu construction in promoting glucose oxidation. The DVC-Cu structure not only increases surface area but also improves mass transfer capabilities, enabling more efficient glucose oxidation reactions at the electrode.

## 4. Conclusions

In this work, we developed DVC-Cu through a straightforward hydrolytic reduction of CuCl, achieving an innovative structure with a Cu^+^/Cu^2+^ interface layer grown onto a preserved CuCl core. The successful synthesis of DVC-Cu was confirmed by XRD, XPS, SEM, and TEM analyses. Compared to its precursor, DVC-Cu demonstrates a unique morphology, higher specific surface area, and reduced impedance, making it ideal for electrochemical applications. The DVC-Cu-based flexible non-enzymatic glucose sensor exhibited high sensitivity (332.4 µA mmol/L^−1^ cm^−2^), wide linear range (up to 10 mmol), and a low detection limit (1.02 nmol/L) in a steady-state test with only 30 µL of electrolyte. The sensor also demonstrated excellent selectivity, stability, and durability against bending and twisting. Additionally, in situ glucose monitoring in human sweat showed a significant current response, highlighting its practical applicability. These results underscore the potential of DVC-Cu as a promising material for developing flexible, non-enzymatic electrochemical sensors for diabetes mellitus type 1 and type 2, such as dynamic real-time glucose concentration monitoring, personalized management, and early warning of diabetes complications.

## Data Availability

The data are not publicly available due to the relevant project regulations.

## Supplementary Materials

The following supporting information can be downloaded at https://www.mdpi.com/article/10.3390/nano14242000/s1, Figure S1: (a) Synthesis illustration and (b) application illustration; Figure S2: SEM-EDS mapping of (a–d) CuCl, and (e–h) DVC-Cu; Figure S3: TEM of (a) CuCl edge, (b) DVC-Cu basement edge; Figure S4: TEM of (a–c) DVC-Cu loaded particles. DVC-Cu outer-shell particles’ (d) size statistics and (e) HRTEM image. (f) electron diffraction of DVC-Cu; Figure S5: CV scanning curves of CuCl, Cu_2_O, DVC-Cu samples at glucose concentrations of 0 mmol/L; Figure S6: CV, IT and ECSA-CV performance of (a–c) CuCl, (d–f) Cu_2_O, (g–i) DVC-Cu; Figure S7: Amperometric response of DVC-Cu based sensor by a stepwise increase of the glucose concentration with (a) +0.5 V, (b) +0.55 V, (c) +0.6 V, (d) +0.65 V, (e) +0.7 V; Figure S8: (a) Amperometric response of DVC-Cu based sensor by a stepwise increase of the glucose concentration at +0.65 V with stirring condition and electrolyte solution was 100 mL mixture of 0.1 mol/L NaOH and 0.1 mol/L NaCl. (b) Relationship between glucose concentration and response current for DVC-Cu material under stirring condition; Figure S9: (a) I-T response while sequentially introducing interfering chemicals alongside 2.0 mmol/L glucose at +0.65 V. (b) I-T response after adding interference agent; Table S1: Glucose electrochemical sensing properties of different potential. (The area of the working electrode is 0.0707 cm^2^); Figure S10: Synthetic human sweat’s amperometric response of DVC-Cu based sensor by a stepwise increase of the glucose concentration while pH was (a) 4.5, (b) 6.5, (c) 8.0, (d) 9.5 and (e) 10.8 at +0.65 V with steady state measurement condition. (f) pH. vs. slop relationship; Figure S11: Synthetic human saliva’s (a) amperometric response of DVC-Cu based sensor by a stepwise increase of the glucose concentration and (b) relationship between glucose concentration and response current; Figure S12: Optical images of (a) intergrade chip, (b) human sweat measurement operation. (c) bending and (d) twisting optical images; Table S2: Glucose electrochemical sensing properties of different electrode materials; Table S3: Glucose determination in human blood serum samples levels (n = 3).
