# Supplementary material for: Dual-Valence Copper Nanostructures with Cu+/Cu2+ Interfaces for High-Sensitivity Glucose Electrochemical Sensing
Source: Nanomaterials (Basel). 2024 Dec 13;14(24):2000. doi: 10.3390/nano14242000 (PMC11728685; doi:10.3390/nano14242000)
Supplement: Supplementary file 1 [file nanomaterials-14-02000-s001.zip › nanomaterials-3338277-supplementary.pdf]

Supplementary Materials

Dual-Valence Copper Nanostructures with Cu^{+}/Cu^{2+} Interfaces for High-Sensitivity Glucose Electrochemical Sensing

Zhipeng Yu[1], Pengxu Yan[1], Yilei Sheng[1], Chengwei Zhang[1], Zhun Qiao[2*], Qikui Fan[1], Chuncai Kong[1], Zhimao Yang[1*]

1 Ministry of Education Key Laboratory for Non-Equilibrium Synthesis and Modulation of Condensed Matter, Shaanxi Province Key Laboratory of Advanced Functional Materials and Mesoscopic Physics, School of Physics, Xi'an Jiaotong University, Xi'an 710049, China

2 Xi'an Rare Metal Materials Institute Co., Ltd., Xi'an 710016, China

* Correspondence: qiaozhun@stu.xjtu.edu.cn (Z.Q.); zmyang@xjtu.edu.cn (Z.Y.)

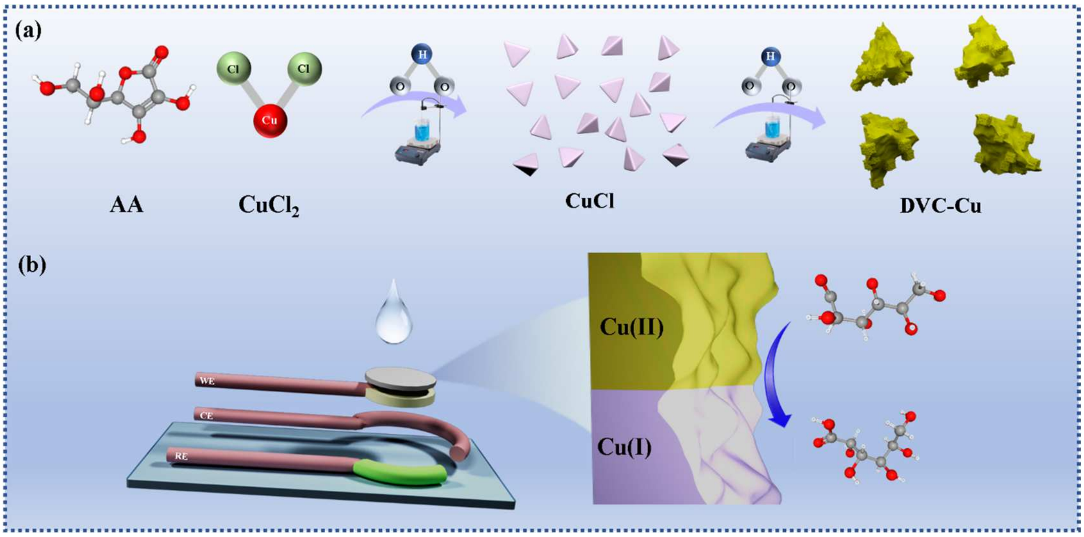

Figure S1. (a) Synthesis illustration and (b) application illustration

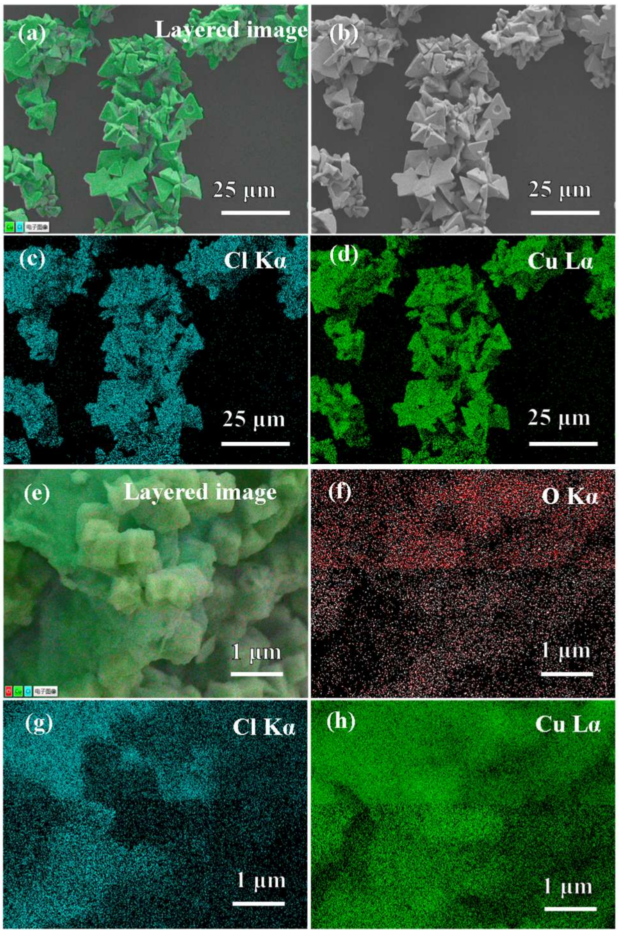

Figure S2. SEM-EDS mapping of **(a, b, c, d)** CuCl, and **(e, f, g, h)** DVC-Cu.

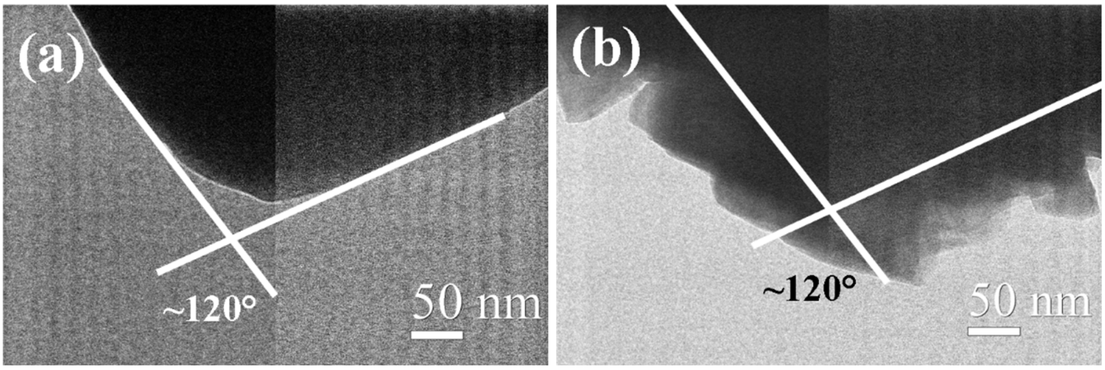

Figure S3. TEM of **(a)** CuCl edge, **(b)** DVC-Cu basement edge.

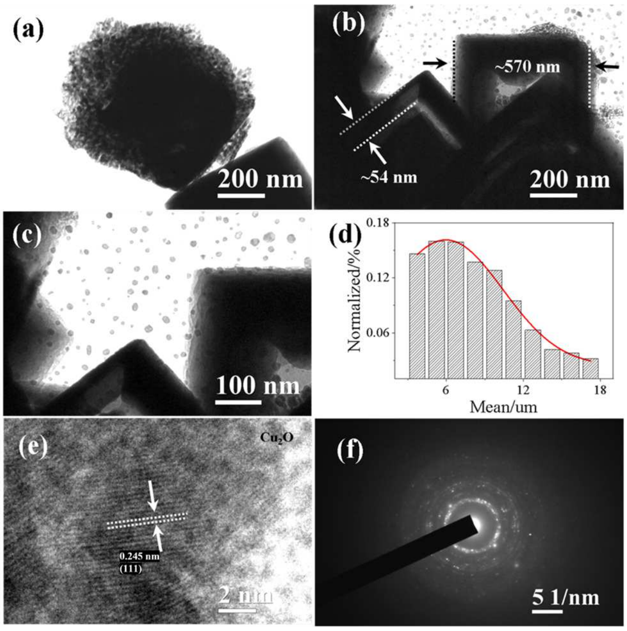

Figure S4. TEM of **(a, b, c)** DVC-Cu loaded particles. DVC-Cu outer-shell particles' **(d)** size statistics and **(e)** HRTEM image. **(f)** electron diffraction of DVC-Cu.

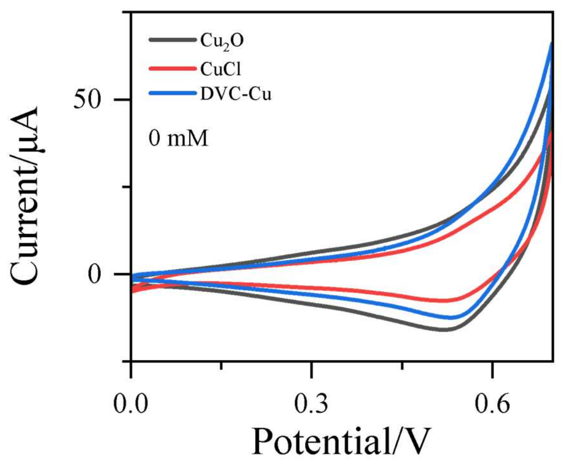

Figure S5.CV scanning curves of CuCl, Cu_2O, DVC-Cu samples at glucose concentrations of 0 mmol/L.

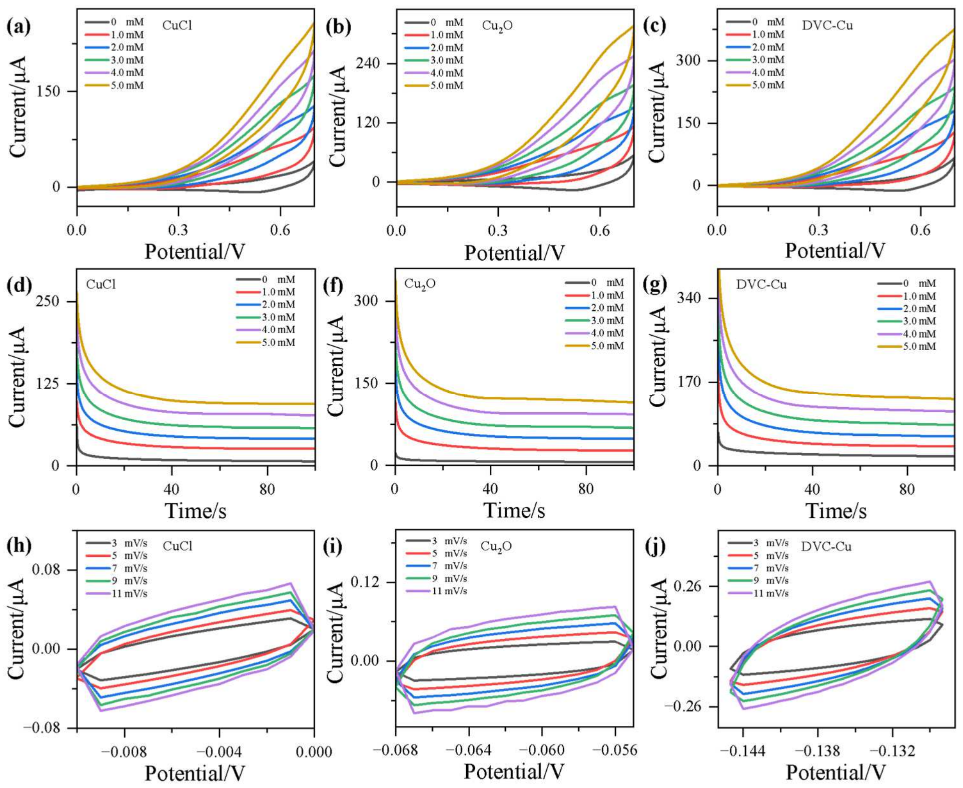

Figure S6. CV, IT and ECSA-CV performance of **(a, b, c)** CuCl, **(d, e, f)** Cu_2O, **(g, h, i)** DVC-Cu.

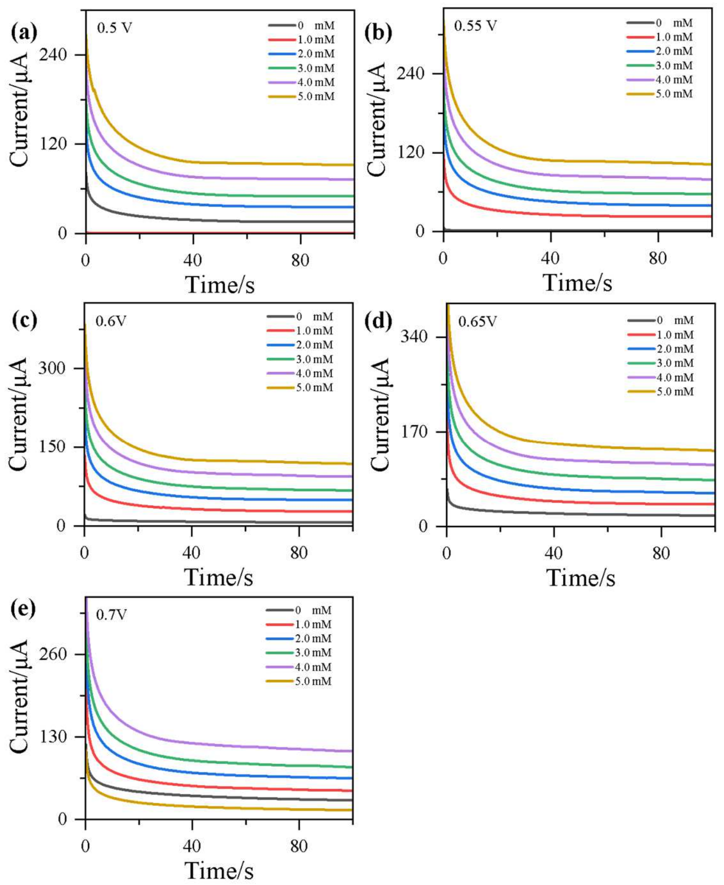

Figure S7. Amperometric response of DVC-Cu based sensor by a stepwise increase of the glucose concentration with **(a)** +0.5 V, **(b)** +0.55 V, **(c)** +0.6 V, **(d)** +0.65 V, **(e)** +0.7 V.

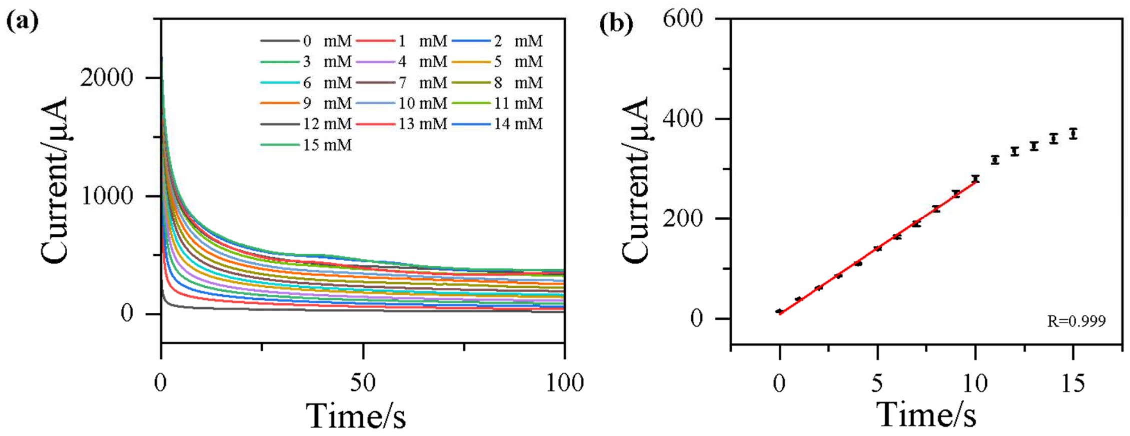

Figure S8. **(a)** Amperometric response of DVC-Cu based sensor by a stepwise increase of the glucose concentration at +0.65 V with stirring conditon and electrolyte solution was 100 mL mixture of 0.1 mol/L NaOH and 0.1 mol/L NaCl. **(b)** Relationship between glucose concentration and response current for DVC-Cu material under stirring condition.

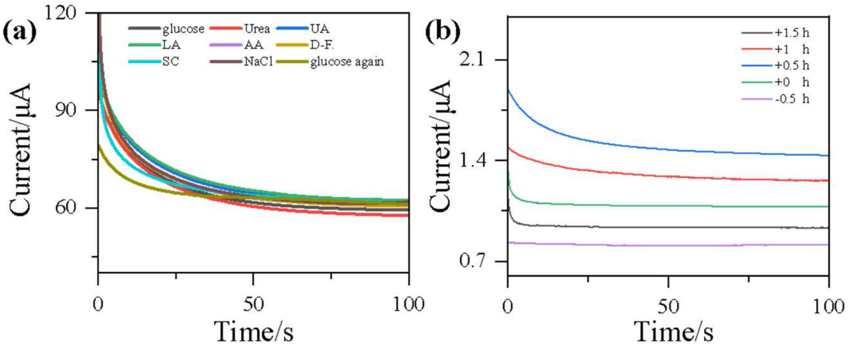

Figure S9. **(a)** I-T response while sequentially introducing interfering chemicals alongside 2.0 mmol/L glucose at +0.65 V.**(b)** I-T response after adding interference agent.

Table S1. Glucose electrochemical sensing properties of different potential. (The area of the working electrode is 0.0707 cm^2)

Potential (V)	Sensitivity ($mAmM^{-1}cm^{-2}$)
0.5	0.2623
0.55	0.2874
0.6	0.3158
0.65	0.3324
0.7	0.260

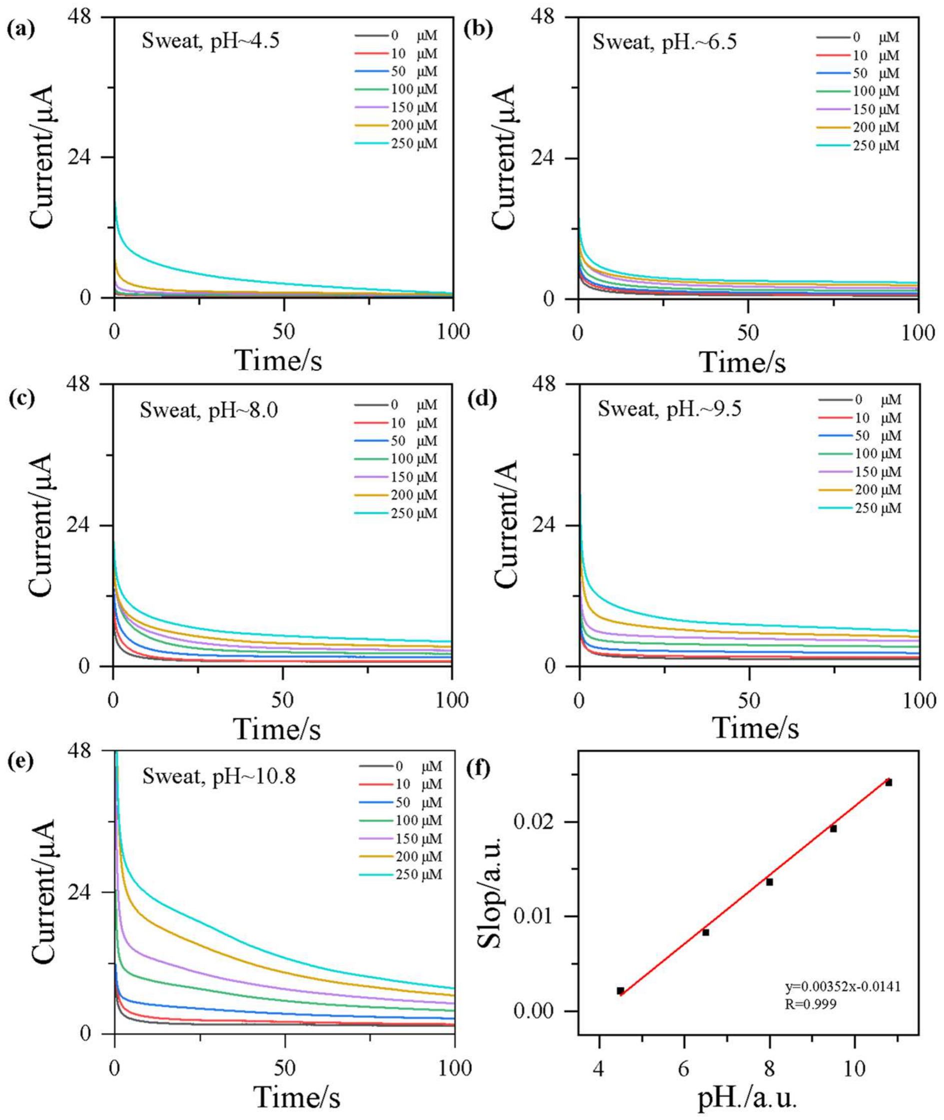

Figure S10. Synthetic human sweat's amperometric response of DVC-Cu based sensor by a stepwise increase of the glucose concentration while pH was **(a)** 4.5, **(b)** 6.5, **(c)** 8.0, **(d)** 9.5 and **(e)** 10.8 at +0.65 V with steady state measurement condition. **(f)** pH. vs. slop relationship.

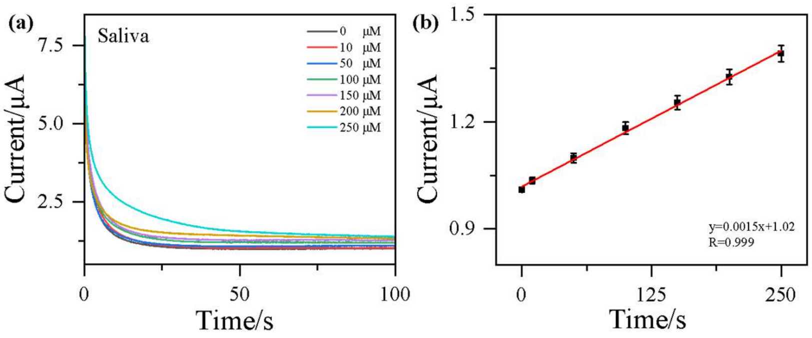

Figure S11. Synthetic human saliva's **(a)** amperometric response of DVC-Cu based sensor by a stepwise increase of the glucose concentration and **(b)** relationship between glucose concentration and response current.

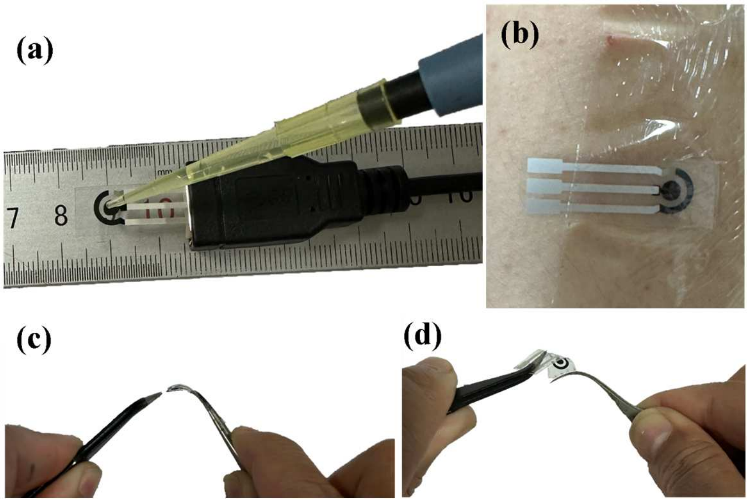

Figure S12. Optical images of **(a)** intergrade chip, **(b)** human sweat measurement operation. **(c)** bending and **(d)** twisting optical images.

Table S2. Glucose electrochemical sensing properties of different electrode materials

	CuCl	Cu_2O	DVC-Cu
Sensitivity ($mAmM^{-1}cm^{-2}$)	0.244	0.301	0.332
LOD (nM)	8.37	2.93	1.02
3n (μA)	0.144	0.0623	0.0239
slop (μA mM^{-1})	17.26	21.29	23.5

Table S3. Glucose determination in human blood serum samples levels. (n=3)

Sample number	R.S.D (.%)	Biochemical analyzer (mmol/L)	Developed glucose sensor (mmol/L)
1	5.52	4.75	4.98
2	3.13	9.25	9.44
3	1.35	11.20	11.31
